# Service environment link and false discovery rate correction: Methodological considerations in population and health facility surveys

**DOI:** 10.1371/journal.pone.0219860

**Published:** 2019-07-18

**Authors:** Teketo Kassaw Tegegne, Catherine Chojenta, Theodros Getachew, Roger Smith, Deborah Loxton

**Affiliations:** 1 Department of Public Health, College of Health Sciences, Debre Markos University, Debre Markos, Ethiopia; 2 Research Centre for Generational Health and Ageing, Hunter Medical Research Institute, School of Medicine and Public Health, University of Newcastle, Newcastle, New South Wales, Australia; 3 The Australian College of Health Informatics, Sydney, New South Wales, Australia; 4 Health System and Reproductive Health Research Directorate, Ethiopian Public Health Institute, Addis Ababa, Ethiopia; 5 Mothers and Babies Research Centre, Hunter Medical Research Institute, School of Medicine and Public Health, University of Newcastle, Newcastle, New South Wales, Australia; Aga Khan University, KENYA

## Abstract

**Background:**

Geospatial data are important in monitoring many aspects of healthcare development. Geographically linking health facility data with population data is an important area of public health research. Examining healthcare problems spatially and hierarchically assists with efficient resource allocation and the monitoring and evaluation of service efficacy at different levels. This paper explored methodological issues associated with geographic data linkage, and the spatial and multilevel analyses that could be considered in analysing maternal health service data.

**Methods:**

The 2016 Ethiopia Demographic and Health Survey and the 2014 Ethiopia Service Provision Assessment data were used. Two geographic data linking methods were used to link these two datasets. Administrative boundary link was used to link a sample of health facilities data with population survey data for analysing three areas of maternal health service use. Euclidean buffer link was used for a census of hospitals to analyse caesarean delivery use in Ethiopia. The Global Moran’s I and the Getis-Ord Gi* statistics need to be carried out for identifying hot spots of maternal health service use in ArcGIS software. In addition to this, since the two datasets contain hierarchical data, a multilevel analysis was carried out to identify key determinants of maternal health service use in Ethiopia.

**Results:**

Administrative boundary link gave more types of health facilities and more maternal health services as compared to the Euclidean buffer link. Administrative boundary link is the method of choice in case of sampled health facilities. However, for a census of health facilities, the Euclidean buffer link is the appropriate choice as this provides cluster level service environment estimates, which the administrative boundary link does not. Applying a False Discovery Rate correction enables the identification of true spatial clusters of maternal health service use.

**Conclusions:**

A service environment link minimizes the methodological issues associated with geographic data linkage. A False Discovery Rate correction needs to be used to account for multiple and dependent testing while carrying out local spatial statistics. Examining maternal health service use both spatially and hierarchically has tremendous importance for identifying geographic areas that need special emphasis and for intervention purposes.

## Background

Geospatial data are important in monitoring many aspects of development; one example of this is in health care. These data are also very useful for informed decision making, providing information on where people and things are located and how people and services interact with each other to produce particular outcomes [1]. Geospatial analysis is a tool that helps to identify gaps and potential barriers to accessing and using health care services. The use of geographic information systems (GIS) allows for the determination of the location of existing health facilities and the target populations for particular health services [2]. In this study, we compare two of these methods by examining the complex issue of maternal health service use in a developing nation.

A geographic linked data analysis requires data on service location, provision and utilization, as well as health outcomes along with location information [3]. Data on service availability and quality, and a facility’s readiness to provide a particular service can be obtained from health facility surveys like the Service Provision Assessment (SPA) [4]. Similarly, population surveys like the Demographic and Health Survey (DHS) provide useful information on a population’s healthcare needs and utilization [5]. A linked analysis of DHS and SPA data enables the examination of the link between population healthcare needs, their utilization and the health service environment [2].

Establishing links between individual health facilities and survey respondents can be done in two different ways. The first approach is mainly dependent on geographic proximity or respondent identification of health facilities visited [6–14]. The second approach links clusters to all health facilities within a certain geographic area, which is useful as the process links every respondent to a service environment [15–18]. Due to the increasing availability of geographically referenced population and health facility data, geographic linking is an efficient approach that maximizes the use of existing data [3, 19]. However, linking these data sources involves critical methodological issues [2, 3]. The two main methodological issues associated with linking the DHS and SPA surveys are geographic displacement and representativeness. Firstly, the SPA surveys, which are stratified samples of public and private health facilities, are not designed to provide statistically representative estimates at the lower geographic levels, for instance, at district levels [20]. Secondly, the geographic locations of each cluster in the DHS survey are displaced before public release for confidentiality issues [21], which is highly associated with misclassification errors [3].

There are four geographic linking methods: sample domain, service environment, estimated surface area and catchment area linkage, which could be used to link DHS and SPA surveys [2]. Each of the above-mentioned methods have their own merits and shortcomings [2], discussion of which is beyond the focus of this paper. For a more detailed discussion about the four geographic linking methods, please see these papers [2, 3]. This paper used the service environment linkage, which links a summary SPA value, such as average service scores, to the DHS clusters [2]. There are several other methods under it: the administrative boundary link, Euclidean buffer link and road network link [2]. To minimize the methodological issues associated with sampled SPA facilities, administrative boundaries (the regional boundaries of Ethiopia) were used. The administrative boundary link is used to link DHS clusters with SPA facilities located within the same administrative boundaries, for instance, located within regions [2, 3]. On the other hand, in case of a census of health facilities, a census of hospitals in this study, Euclidean buffer link was used. The Euclidean buffer link uses a buffer distance from cluster centroid to health facility, which then links every hospital located within the buffer without considering administrative boundaries [2, 3].

Healthcare seeking and use, particularly maternal health services, are context specific and multilevel in nature [22, 23]. Socio-cultural, geographic, economic and political factors play a significant role in the use of maternal health services. Governments’ healthcare investment and expenditure has a significant effect in improving women’s use of maternal health services [24, 25]. Geographic access, which could be measured in terms of distance and/or travel time, is also one of the most important factors in accessing and using maternal healthcare services [26]. Furthermore, availability and quality of services, and a facility’s readiness to provide these services are very important for maternal health service use [27]. Similarly, individual and community level factors such as media, education and wealth were significantly associated with utilization of maternal health services [23, 28–30].

This paper addressed the methodological issues associated with three areas of maternal health services, namely family planning, antenatal and delivery care, including caesarean delivery. The paper aimed to compare the geographic linking methods used for both a sample and a census of health facilities. Especially, it compared the two direct geographic linking methods: administrative boundary link and Euclidean buffer link, for linking geographically referenced household and health facility data. Furthermore, it explored the spatial and multilevel analysis that could be used for maternal health services after linking these datasets.

## Methods

### Data sources

Data from the Democratic Republic of Ethiopia were used for this analysis. The DHS and SPA surveys, which were conducted within a 19-month window, were used. Geographic coordinates were available for both datasets.

#### 2016 Ethiopia Demographic and Health Survey (EDHS)

The 2016 EDHS used the 2007 Ethiopian Population and Housing Census sampling frame. The census frame has a list of 84,915 Enumeration Areas (EAs) that were prepared for the 2007 national census [31]. In general terms, an EA is a geographic location that has an average of 181 households. Each sampling frame has information on EA location, residence (rural or urban) and the estimated number of households. The 2016 EDHS survey was a cross-sectional household study; it is the main source of data on population healthcare utilization. The survey used a stratified sampling procedure in two stages. Urban and rural area stratification was made for each region which yielded 21 sampling strata [31].

At stage one, 645 EAs (202 versus 443 in urban and rural areas, respectively) were sampled using a probability proportional to enumeration size. Before the actual data collection, a list of households was made in the sampled EAs. At stage two, households were selected using a systematic sampling technique from the list of households in each of the EAs. A fixed number of 28 households were sampled per EA using an equal probability allocation. All women aged 15–49 years were eligible for individual interviews. A total of 15,683 women of reproductive age were interviewed out of the identified 16,583 eligible women [31].

#### 2014 Ethiopia Service Provision Assessment Plus (ESPA+)

The 2014 ESPA+ survey was a health facility-based cross-sectional study, and is the main source of data on the availability of health services. This survey used a list of 23,102 formal health facilities operating in the country. The list was obtained from the Federal Ministry of Health. Two hundred and two hospitals, 3,292 health centres, 15,618 health posts and 3,990 clinics (higher, medium and lower clinics) were included in the list. These facilities were managed by the government, private for profit and non-governmental organizations. A combination of census and simple random sampling techniques were used to select health facilities [32].

Because of their importance and limited numbers, all hospitals, with the inclusion of all newly identified hospitals, were included in the survey. However, a representative sample of health centres and clinics was selected from a master health facility list. Health posts were selected independently. In total, 1,327 health facilities, which includes 321 health posts and 10 newly identified hospitals, were included in the survey. Due to various reasons (security issues in Somali region, inability to obtain consent at military hospitals, and duplicate facility names), data were collected from 1,165 health facilities representing 88% of sampled facilities [32].

### Data linking methods

The EDHS provides data on utilization of health services as well as respondents’ socio-demographic characteristics, while the ESPA+ survey provides information on service availability and facilities’ readiness to provide services. The geographic coordinates and region identification codes collected in both surveys were used to link each DHS cluster and SPA facility score. Clusters and health facilities with missing geographic coordinates were excluded. The administrative polygons of Ethiopia, which were obtained from Natural Earth [33], were also used. Two geographic linking methods were used for directly linking clusters with health facilities: administrative boundary link and Euclidean buffer link.

#### Administrative boundary link

In Ethiopia, family planning, antenatal and delivery care services are being provided at all levels of health facilities, such as at health posts, clinics, health centres and hospitals. However, with the exception of a census of hospitals, the SPA survey collected these data from sampled health facilities. In this case, using geographic linking method to link sampled health facilities with DHS clusters is challenging. For instance, geographic linking based on the nearest sampled health facility would be problematic as the nearest health facility to each DHS cluster might not be included in the SPA survey, which could result in misclassification error. On this occasion, an administrative boundary link is an appropriate choice for directly linking sampled health facility data with DHS survey data [2, 3]. This method links all DHS clusters with all health facilities found within the respective administrative boundaries. In this study, city administrations and administrative regions of Ethiopia were used as administrative boundary link. This data linking approach was used for contraceptive, antenatal care and health facility delivery data analysis. As a result, this method did not miss any health facility that falls within the respective administrative boundaries.

#### Euclidean buffer link

In Ethiopia, caesarean delivery is being provided at emergency obstetric care (EmOC) facilities. The SPA survey collected data from all hospitals. In this case, geographically linking census health facilities with DHS clusters provides a good picture of service environment at cluster level. Euclidean buffer link is used for linking a census of health facilities data with population survey data [2, 3]. This method links all EDHS clusters with all hospitals found within a defined buffer distance; in this case, the closest hospital to each cluster was linked. This approach was used for caesarean delivery analysis. This method avoids an unnecessary merging of health facilities that can result in loss of information at cluster level, which is the shortcoming of administrative boundary link.

### Health service environment and measurements

The links between SPA facilities and DHS clusters were defined by creating healthcare service environment variables. In this analysis, maternal health facilities are defined as any healthcare facility providing family planning, antenatal care, and basic and comprehensive obstetric care services. The following four health service environment variable scores, taken from the SPA survey, were created: average distance to the nearest maternal health facilities, maternal health service availability score, readiness to provide maternal health services score, and a general health facilities readiness score. The maternal health indices (family planning, antenatal care, basic obstetric care and comprehensive obstetric care indices) were created using the World Health Organization’s ‘Service Availability and Readiness Indicators’ [34, 35].

Average distance to the nearest maternal health facility was calculated after linking each DHS cluster with the SPA facilities. PROC SQL was used to link the two data sets using their geographic coordinates in SAS. Since the SPA facilities, except a census of hospitals, were sampled, taking the nearest health facility to each cluster would have been problematic. In the ESPA survey, for instance, the nearest health facility to every EDHS cluster might not have been included. Therefore, to have a representative distance across the nine regions and the two city administrations, regional average distances were calculated for contraceptive, antenatal care and health facility delivery data analysis. On the other hand, since all the hospitals were included in the SPA survey, the nearest hospital providing caesarean delivery in each cluster was used for caesarean delivery analysis.

A principal component analysis was used to compute all service availability and readiness scores for health facilities. General service readiness for all health facilities was computed. Six general service readiness dimensions were used for caesarean delivery, eight for family planning, and nine for both antenatal care and health facility delivery services. For each maternal health service, the first two principal components (health facility management system and infrastructure) in the principal component analysis were used to compute two general service readiness scores.

With regard to service specific scores, for those health facilities which reported as providing family planning services, indices of family planning availability and readiness were created. Family planning availability scores were created using seven variables (combined oral contraceptive pills, progestin-only contraceptives pills, progestin-only injectable contraceptives, intrauterine device, emergency contraceptive pills, male sterilization, and female sterilization). For each indicator, in order to measure the availability of family planning services, health facilities were given one point for services available and zero for unavailable services. Thus, two family planning availability scores (long acting and short-term contraceptives methods) were created using the principal component analysis. Similarly, a family planning service readiness score was computed using seven dichotomous variables (family planning training, family planning checklists and/ or job-aids, combined oral contraceptive pills, progestin-only injectable contraceptives, intrauterine device, implants, and emergency contraceptive pills). The principal component analysis resulted in two family planning readiness scores (readiness to provide long acting and short-term contraceptives) that were used to measure a facility’s readiness to provide family planning services.

For antenatal care providing facilities, indices of antenatal care availability and readiness were created. One antenatal care availability score (antenatal care supplements) was created using four variables (iron and folic acid supplements, tetanus toxoid vaccination, and a combination of iron and folic acid supplements). Similarly, an antenatal care service readiness score was computed using six dichotomous variables (ANC guideline, ANC checklists and/or job aids, staff trained in ANC service, urine dipstick/protein and haemoglobin test, and tetanus toxoid vaccine). The principal component analysis resulted in two antenatal care readiness scores (readiness to provide diagnostic services and skilled care), which were used to measure a facility’s readiness to provide antenatal care services.

Furthermore, for those health facilities reported as providing basic obstetric care services, indices of basic obstetric care availability and readiness were created. Basic obstetric care availability score was created using seven variables (parenteral administration of antibiotics, parenteral administration of uterotonic drugs, parenteral administration of anticonvulsants, assisted vaginal delivery, manual removal of placenta, manual removal of retained products, and neonatal resuscitation). For each indicator, in order to measure the availability of basic obstetric care services, health facilities were given one point for services available and zero for unavailable services. Thus, one basic obstetric care availability score (BEmOC signal functions) was created using the principal component analysis. Similarly, a basic obstetric care readiness score was computed using twelve dichotomous variables (staff trained in delivery & newborn care, skilled delivery care provider [24 hour coverage], examination light, delivery pack, suction apparatus [mucus extractor], manual vacuum extractor, vacuum aspiration [D&C kit], neonatal bag and mask, blank partograph, antibiotic eye ointment for newborn [e.g., Tetracycline], injectable antibiotic [e.g., Ceftriaxone], and IV solution [Ringer lactate & Normal saline] with infusion set). This analysis resulted in three basic obstetric care readiness scores (skilled personnel, medicine and commodities, and delivery equipment) that were used to measure a facility’s readiness to provide basic obstetric care services.

Indices of comprehensive obstetric care availability and readiness were created for comprehensive obstetric care providing facilities. Two comprehensive obstetric care availability scores (basic and comprehensive components) were created using seven variables (parenteral administration of antibiotics, parenteral administration of uterotonic drugs, parenteral administration of anticonvulsants, assisted vaginal delivery, manual removal of retained products, neonatal resuscitation and blood transfusion). Comprehensive obstetric care readiness scores were computed using nine dichotomous variables (staff trained in delivery & newborn care, anaesthesia equipment, resuscitation table or neonatal resuscitation kit, oxygen, Spinal needle, blood typing, cross match testing, blood supply sufficiency, and caesarean section set). The analysis resulted in two comprehensive obstetric care readiness scores (equipment and supplies, and skilled personnel) that were used to measure a facility’s readiness to provide comprehensive obstetric care services.

With regard to measuring outcome variables, a woman was considered to be using modern contraception if she used any modern contraceptive methods including female sterilization, male sterilization, oral contraceptive pills, intrauterine device (IUD), injectables, implants, or the lactational amenorrhea method. Male condom use was excluded since women could obtain condoms from shops that the SPA survey did not capture. For the antenatal care analysis, a woman’s use of antenatal care for her most recent birth in the five years preceding the survey was measured based on the number of antenatal visits. Pregnant women were grouped into three categories: those who had no ANC visits; one to three ANC visits; and four or more ANC visits. Regarding health facility delivery, a pregnant woman was considered to be using facility delivery if she reported that her most recent birth (within the five years preceding the survey) was at a health facility. Lastly, a woman was considered to have used caesarean delivery if her most recent birth (within the five years preceding the survey) was via caesarean section.

### Statistical analysis

The two data sets were linked using SAS software. The spatial analysis can be carried out using ArcGIS software. The Ethiopian Polyconic Projected Coordinate System, based on the World Geodetic System 84 (WGS84) coordinate reference system (CRS), was used to produce a flattened map of the country. The spatial statistics can be used to identify statistically significant spatial clusters (hot/cold spots) of maternal health service use. The GLIMMIX procedure in SAS can be used to estimate hierarchical models for categorical data, in this case, maternal health service use.

#### Spatial analysis: Global Moran’s I statistic

The Global Moran’s I statistic or global spatial autocorrelation is the first step to be carried out in identifying spatial patterns of observations. It is used to measure the overall clustering and test the null hypothesis that there was complete spatial randomness (no spatial clustering) of observations [36]. It is used to measure the correlation between neighbouring observations and to find out spatial patterns and level of spatial clustering among neighbouring features [37]. The Global Moran’s I statistic is calculated by [38]:

$$I=\frac{n}{S_{O}}\sum_{i}\sum_{j}w_{ij}\frac{\left(x_{i}-\bar{x})(x_{j}-\bar{x}\right)}{\sum_{i}{\left(x_{i}-\bar{x}\right)}^{2}}$$

where *n* is the number of features (it is the number of clusters in this study), *w*_*ij*_ is the spatial weight between feature *i* and *j*, *x*_*i*_ and *x*_*j*_ are attribute values for feature *i* and *j*, respectively with mean $\bar{x}$ and *S*_*o*_ is the aggregate of all the spatial weights: ***S***_***o***_ = ∑_***i***_∑_***j***_***w***_***ij***_

The *Z*_*I*_*-score* for the statistic is computed as $Z_{I}=\frac{I-E[I]}{\sqrt{Var[I]}}$ where ***E***[***I***] = −**1**/(***n***−**1**) and ***Var***[***I***] = ***E***[***I***^**2**^]−***E***[***I***]^**2**^

However, the Global Moran’s I (the measure of overall spatial autocorrelation) answers only the question “Is there spatial clustering?”; it does not answer the question “Where are the clusters (hot spots/cold spots)?”[39]. Therefore, local measures of spatial autocorrelation, that is Hot Spot Analysis (Getis-Ord Gi* statistic), are necessary to identify the type of spatial correlation and test the significance of local spatial patterns.

#### Spatial analysis: Incremental spatial autocorrelation

The next step in identifying spatial clusters is to carry out incremental spatial autocorrelation. The incremental spatial autocorrelation is important to determine the scale, that is the critical distance or distance bandwidth at which there is maximum clustering. It measures spatial autocorrelation for a series of distances and creates a line graph with corresponding z-scores. The z-scores reflect intensity of spatial clustering; statistically significant z-scores indicate the distances at which maximum clustering are pronounced [40]. Before running the incremental spatial autocorrelation, the average distance at which a feature has at least one neighbour needs to be calculated using the *Calculate Distance Band from Neighbour Count* in the Spatial Statistics tools toolbox in ArcMap. Then, the maximum distance at which clustering of maternal health service use peaked can be obtained with corresponding z-score after running the incremental spatial autocorrelation.

#### Spatial analysis: Getis-Ord Gi* statistic

Lastly, the Getis-Ord Gi* statistic uses this maximum distance to identify statistically significant spatial clusters of hot spots (areas of high maternal health service use rates) and cold spots (low maternal health service use rates). The Getis-Ord Gi* statistic (local G-statistic) is used to test the statistical significance of local clusters and to determine the spatial extent of these clusters [41]. It is useful for identifying clusters by determining spatial dependence and relative magnitude between an observation and its neighbouring observations. The Getis-Ord local statistics [42] is given as:

$$G_{i}^{*}=\frac{\sum_{j=1}^{n}w_{ij}x_{j}-\bar{x}\sum_{j=1}^{n}w_{ij}}{S\sqrt{\frac{\left[n\sum_{j=1}^{n}w_{ij}^{2}-{\left(\sum_{j=1}^{n}w_{ij}\right)}^{2}\right]}{n-1}}}$$

where *x*_*j*_ is the attribute value for feature *j*, *w*_*ij*_ is the spatial weight between feature *i* and *j*, *n* is equal to the total number of features and $\bar{x}$ is the mean maternal health service use: $\bar{x}=\frac{\sum_{j=1}^{n}x_{j}}{n}$ and $S=\sqrt{\frac{\sum_{j=1}^{n}x_{j}^{2}}{n}-{\left(\bar{x}\right)}^{2}}$

The $G_{i}^{*}$ statistic is a z-score, which means no further calculations are required. The $G_{i}^{*}$ is assumed to be normally distributed [41]. In other words, it can be calculated as a standard normal variant with a probability from the *z-score* distribution [43]. Clusters with a 95% significance level from a two-tailed normal distribution indicate significant clustering. A z-score of near zero and p-value greater than 0.05 indicate complete spatial randomness within the study area. A positive z-score along with p-value less than 0.05 indicate clustering of high values.

#### Spatial analysis: False discovery rate correction

Assessing the significance of local statistics of spatial association gets more complex as the number of spatial features/locations increases. In spatial analysis, it is fundamentally important to account for multiple and dependent comparisons. A False Discovery Rate (FDR) correction method can be applied to account for multiple and dependent tests in Local Statistics of Spatial Association [44]. A comparison of local statistic results was made with and without applying the False Discovery Rate correction in ArcGIS.

#### Multilevel analysis

After linking women in the respective cluster to the health facility variables, a multilevel regression analysis can be carried out. The EDHS survey employed a multistage cluster sampling technique where women in the survey were nested within regions. Due to the hierarchical nature of the data, analysis can be done using a two stage Hierarchical Generalized Linear Model (HGLM), which is appropriate for categorical, non-normally distributed response variables including binary data. The GLIMMIX procedure (PROC GLIMMIX) in SAS can be used to estimate the hierarchical generalized linear models [45].

The equation necessary for estimating this two level model is presented below.

$$Y_{ij}=\gamma_{00}+\gamma_{01}\;W_{j}+\mu_{0j}+\gamma_{10}X_{ij}+\mu_{1j}X_{ij}$$

where ***Y***_***ij***_ represents the log odds of using maternal health service for woman ***i*** in region ***j***, ***γ***_**00**_ provides the log odds of using maternal health service in a typical region, ***W***_***j***_ is a region-level predictor for region ***j***, ***γ***_**01**_ is the slope associated with this predictor, ***μ***_**0*j***_ is the level-2 error term representing a unique effect associated with region ***j*, *γ***_**10**_ is the average effect of the individual-level predictor, ***X***_***ij***_ is an individual-level predictor for woman **i** in region ***j***, and ***μ***_**1*j***_ is a random slope for a level-1 predictor variable ***X***_***ij***_, which allows the relationship between the individual-level predictor (***X***_***ij***_) and the outcome (***Y***_***ij***_) to vary across level-2 units.

This analysis procedure enabled the identification of potential factors associated with the utilization of maternal health service with a 95% confidence interval and p-value < 0.05. A common maximum likelihood estimation technique available with PROC GLIMMIX in SAS (the Laplace estimation) can be used to estimate the best-fit model [45]. The model building process should start with the unconditional model (a model containing no predictors) and more complex models can be gradually built by checking improvements in model fit after each model is estimated. A likelihood ratio test that examines the difference in the -2 log likelihood (-2LL) can be used to assess the best fitting model [45]. The unconditional (empty) model is used to calculate the intra-class correlation coefficient (ICC), which estimates how much variation in the use of maternal health service exists between regions (level-2 units). In HGLMs, it is assumed that there is no level-1 error variance; to calculate the intra-class correlation coefficient, a slight modification is made. The level-1 residual variance (***ε***_***ij***_**)** follows a logistic distribution and is standardized with a mean of zero and variance = π23 [46]. Therefore, for a two–level random intercept HGLM with an intercept variance of $\sigma_{\mu 0}^{2}$, the intra-class correlation coefficient (Rho) is given by; ρ=σμ02σμ02+π23 [46].

### Ethics approval

Ethical approval was obtained from the Human Research Ethics Committee, The University of Newcastle. We also got the Ethiopian Public Health Institute (EPHI) and the Measure DHS program approval to access the datasets.

## Results

### Administrative boundary link versus Euclidean buffer link

In the case of administrative boundary link, it was found that all of the maternal health service providing SPA facilities were linked to DHS clusters. Similarly, more types of SPA facilities, such as hospitals and health centres were linked to the DHS clusters. For instance, all of the family planning providing hospitals and health centres were linked to the DHS clusters. The Euclidean buffer link, on the contrary, gave a small number of health facilities providing maternal health services. For example, only 44.31% and 47.23% of family planning and antenatal care providing facilities were linked to DHS clusters, respectively. This link also gave the least number of family planning providing facility types; for instance, only 40% of family planning providing clinics were linked to DHS clusters. On the other hand, for a census of hospitals, the Euclidean buffer link (the nearest hospital to each DHS cluster) gave an excellent link. Out of the 179 caesarean delivery-providing hospitals, 81.56% of them were linked to the DHS clusters (Table 1).

**Table 1:** Number (percent) of SPA facilities linked to DHS clusters by linking method.

Geographic linking method	Administrative boundary link		Euclidean buffer link (Nearest)	
	SPA facilities			
	No	%	No	%
**Type of service each health facility providing**				
Family planning	1020	100	452	44.31
Antenatal care	919	100	434	47.23
Delivery care	717	100	389	54.25
Caesarean delivery	179	100	146	81.56
**Facility type (family planning providing facilities)**				
Hospital	191	100	79	41.36
Health centre	283	100	143	50.53
Health post	271	100	120	44.28
Clinic	275	100	110	40.00

### False Discovery Rate (FDR) correction

Without adjusting for multiplicity and spatial dependence–without applying FDR correction while running local spatial statistics as shown in Table 2, a higher number of false positive clusters were identified. However, when the FDR correction was applied, the total number of clusters identified as either hot spot or cold spot was decreased. For example, with the 95% confidence interval, 169 clusters were identified as hot spots of modern contraceptive use when controlled for FDR as compared to the 199 clusters of high modern contraceptive use areas without FDR correction. On the other hand, a higher number of DHS clusters (367) were not found significant with controlled FDR as compared to the 272 non-significant clusters in the use of modern contraceptive without applying FDR correction. These differences were also observed in the case of the other maternal health services use, such as having at least four ANC visits and the use of caesarean delivery (Table 2).

**Table 2:** Comparison of hot spot analysis results with and without FDR correction.

95% Confidence interval	Without FDR correction			With FDR correction		
	Modern contraceptive use	At least 4 ANC visits	Caesarean delivery	Modern contraceptive use	At least 4 ANC visits	Caesarean delivery
Cold spot	151	70	221	86	68	167
Not significant	272	486	198	367	537	253
Hot spot	199	66	203	169	17	202

**Fig 1:**
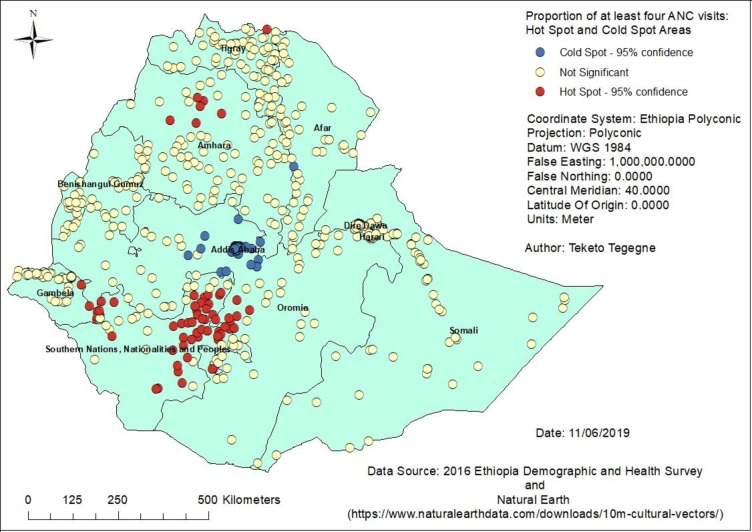
Clusters of at least four ANC visits in Ethiopia without FDR correction, 2016.

**Fig 2:**
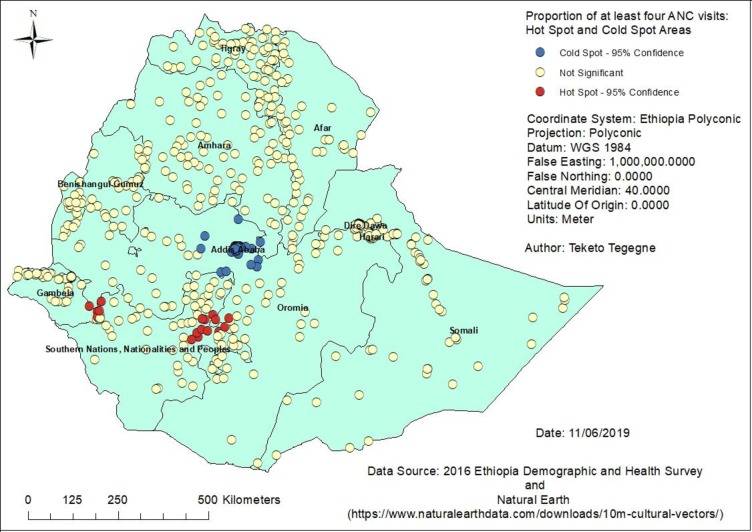
Clusters of at least four ANC visits in Ethiopia with FDR correction, 2016.

These differences were also easily observed from the hot spot analysis map. For showing the differences in how the FDR correction identifies true clusters in comparison to uncontrolled clusters for multiple and spatial dependence, only antenatal care visits maps are presented in this paper (Figs 1 and 2).

## Discussion

A linked spatial data analysis provides important information on the different types and quality of health services accessed and factors related to healthcare-seeking behaviours [2]. It also provides important information for program managers and decision makers on health facilities and services as well as information about the communities that need special attention [47, 48]. The analysis can also be used for program monitoring and evaluation [2]. Mapping of the combined findings of service utilization and health facility distribution is very useful for a better understanding of a particular situation, planning programs and services, advocacy, and creating awareness of a particular phenomenon [48].

In this paper we explained the service environment link, which is the most appropriate geographic linking method for both a sample and a census of health facilities [2, 3]. Most of the methodological issues associated with linking geographic data are minimized. The SPA samples, with the exception of hospitals, were not well represented at the lowest level of geographic disaggregation [20]. To minimize this issue, administrative boundary link was applied. It is the least to be affected by issues of facility sample and geographic displacement [3]. However, this approach results in a loss of an enormous amount of information that is due to merging of facility level information to administrative boundary levels. It is difficult to get cluster level service environment estimates to run a regression analysis. In this case, administrative level service environment estimates are the appropriate choice to run a regression analysis.

The other methodological issue is temporal difference between surveys [3]. Due to the rapid changes in the healthcare environment, such as availability of contraceptive commodities, linking these two datasets could introduce measurement errors. However, this paper used two surveys conducted within a 19-month window that could minimize temporal differences in the healthcare service environment. In addition to this, since the population survey captured the preceding five years’ individual service utilization covered by the SPA survey, this could largely minimize the temporal differences in the healthcare environment.

Misclassification errors related to geographic data are the other concern of geographic data linkage. First, this paper did not consider topological features, such as mountains, that could impede access to healthcare facilities [49]. These features could potentially affect access to maternal health facilities where the average distances were defined based on a straight-line distance. Second, in the case of a census of health facilities (hospitals, in this study), the misclassification errors associated with DHS geographic coordinate displacement [3, 21] could change the actual cluster distance to nearby hospitals, even if large buffer distances are used. Third, there could also be a misclassification error related to bypassing the nearest healthcare facility [50]. However, this paper used regional administrative boundaries and regional average distances to minimize errors related to bypassing a particular healthcare facility. Furthermore, the Euclidean buffer link avoids an unnecessary merging of facility level information and enables the provision of cluster level service environment estimates. For instance, in the case of caesarean delivery, it enables us to estimate how the nearby hospital’s readiness to provide caesarean delivery affects pregnant women’s use of this particular service.

Tobler’s First Law of Geography states that, *“Everything is related to everything else*, *but near things are more related than distant things”* [51]. This law applies in healthcare phenomena which have a spatial nature. For instance, geographic inequity in maternal and child health outcomes is a significant issue at various levels. Similarly, the location of health services is a key factor for accessing and using a particular care [52]. The statistical analysis of these spatial phenomena is called spatial data analysis [53]. It is very important for identifying spatial distribution, pattern and occurrence of spatial outliers of a phenomenon [54]. It is a powerful analysis for identifying areas with spatial disparity, aiding resource allocation and intervention, which are important for effective healthcare programming [55, 56].

Measures of spatial dependence (spatial autocorrelation) can be done at global or local level. The global measures give a single statistic summarizing the spatial pattern by using all the available locations simultaneously. However, local statistics measure the spatial association between each location and its neighbor based on defined distances. It gives one statistic for every location enabling identification of clusters [57]. This is the most important component of spatial analysis for any measures or interventions to be undertaken such as setting up new hospitals.

However, doing a local statistic has two main statistical issues. Since local statistics depend on tests of spatial association for each location, multiple comparison is a concern in assessing statistical significance [58, 59]. When doing multiple comparison, selection of statistically significant differences is a critical issue. One way to select statistically significant effect or difference is by not using multiple comparison methods. However, this inflates Type I error, which is the probability of rejecting one or more null hypotheses when the likelihood of each one actually being true is high. Therefore, in this case, false clusters of phenomena are likely to be identified. The Bonferroni and Sidak corrections are highly conservative multiple comparison procedures [36, 57, 60]. The Bonferroni adjustment is simple and trustworthy for ensuring that the probability of any single Type I error cannot be greater than α. Nevertheless, this can partially identify clusters and result in the missing of many true clusters.

In addition to the multiple comparison issue, local statistics are calculated based on a defined neighborhood size where locations containing common neighbors are likely to be correlated [36, 57, 61]. A correction for spatial dependence to conservative methods, a method that accounts for common neighbors shared by nearby locations, can be applied [60]. However, using a correction procedure that accounts for both multiple comparison and dependence test is a good solution. This paper used the False Discovery Rate correction, which is a powerful method that accounts for both multiple and spatial dependence tests. The FDR correction fully identifies true clusters and improves spatial dependence test results as compared to the other methods [44].

Hierarchically organized data are common in healthcare research. For example, the Ethiopian Demographic and Health Survey (EDHS) employed a multistage cluster sampling technique where survey respondents were nested within clusters and clusters were nested within regions [31]. Conducting research that ignores a level of nesting in data can lead to erroneous conclusions. This can affect estimated variances and the power to detect covariate effects [62, 63], inflate Type I error rates [64] and introduce errors in interpreting statistical significance test results [65, 66]. Thus, multilevel models that are developed to account for hierarchical nesting of data [67–69] are very important statistical solutions. It provides rich information about how things operate at different levels of hierarchy, for instance, maternal health service use. By including random effects in the model, it enables the identification of multiple sources of variation in the use of maternal healthcare services.

## Conclusion

A service environment link is the method of choice for linking a sample and a census of health facility data with population surveys. This minimizes the methodological issues associated with geographic data linkage. In case of sampled health facilities, administrative boundary link is the method of choice whereas the Euclidean buffer link is the appropriate choice for a census of health facilities. The Euclidean buffer link enables the provision of cluster level service environment estimates as opposed to the administrative boundary link. Examining maternal health service use spatially has tremendous importance for identifying geographic areas that need special attention and intervention purposes. A False Discovery Rate correction needs to be considered to account for multiple and spatial dependence while carrying out local spatial statistics. Moreover, considering hierarchical nature of data in healthcare research is important for identifying key determinants of health service use.

### Ethics approval and consent to participate

Ethical approval was obtained from the Human Research Ethics Committee, The University of Newcastle. We also got the Ethiopian Public Health Institute (EPHI) and the Measure DHS program approval to access the datasets.

## Abbreviations

ANC: Antenatal Care
BEmOC: Basic Emergency Obstetric Care
CRS: Coordinate Reference System
DHS: Demographic and Health Survey
EAs: Enumeration Areas
EDHS: Ethiopia Demographic and Health Survey
EmOC: Emergency Obstetric Care
ESPA+: Ethiopia Service Provision Assessment Plus
FDR: False Discovery Rate
GIS: Geographic Information Systems
HGLM: Hierarchical Generalized Linear Model
SPA: Service Provision Assessment
WGS84: World Geodetic System 84
WHO: World Health Organization

## References

[1] United Nations. The Millennium Development Goals Report 2015. New York: United Nations; 2015.

[2] Burgert-Brucker CR, Prosnitz D. Linking DHS household and SPA facility surveys: Data considerations and Geospatial Methods. DHS Spatial Analysis Reports No. 10: ICF International; 2014.

[3] Skiles MP, Burgert CR, Curtis SL, Spencer J. Geographically linking population and facility surveys: methodological considerations. Population health metrics. 2013;11(1):14 10.1186/1478-7954-11-14 23926907 PMC3765268

[4] Hozumi D, Fronczak N, Minichiello SN, Buckner B, Fapohunda B, Kombe G, et al Profiles of Health Facility Assessment Methods Report of the International Health Facility Assessment Network (IHFAN). MEASURE Evaluation, USAID; 2008.

[5] Wang W, Winter R, Mallick L, Florey L, Burgert-Brucker C, Carter E. The Relationship between the Health Service Environment and Service Utilization: Linking Population Data to Health Facilities Data in Haiti and Malawi DHS Analytical Studies No. 51. Rockville, Maryland, USA: ICF International; 2015.

[6] Kashima S, Suzuki E, Okayasu T, Louis RJ, Eboshida A, Subramanian S. Association between proximity to a health center and early childhood mortality in Madagascar. PLoS ONE. 2012;7(6):e38370 10.1371/journal.pone.0038370 22675551 PMC3366931

[7] Gabrysch S, Cousens S, Cox J, Campbell OM. The influence of distance and level of care on delivery place in rural Zambia: a study of linked national data in a geographic information system. PLoS medicine. 2011;8(1):e1000394 10.1371/journal.pmed.1000394 21283606 PMC3026699

[8] Schoeps A, Gabrysch S, Niamba L, Sié A, Becher H. The effect of distance to health-care facilities on childhood mortality in rural Burkina Faso. Am J Epidemiol. 2011;173(5):492–8. 10.1093/aje/kwq386 21262911

[9] Målqvist M, Sohel N, Do TT, Eriksson L, Persson L-Å. Distance decay in delivery care utilisation associated with neonatal mortality. A case referent study in northern Vietnam. BMC Public Health. 2010;10(1):762. 21144058 10.1186/1471-2458-10-762 PMC3009650

[10] Heard NJ, Larsen U, Hozumi D. Investigating access to reproductive health services using GIS: proximity to services and the use of modern contraceptives in Malawi. African Journal of Reproductive Health. 2004:164–79. 15623130

[11] Chen SCC, Wang JD, Yu JKL, Chan CC, Nyasulu YM, Kolola‐Dzimadzi R. Applying the global positioning system and google earth to evaluate the accessibility of birth services for pregnant women in northern Malawi. Journal of midwifery & women's health. 2011;56(1):68–74. 10.1111/j.1542-2011.2010.00005.x 21323853

[12] Anson O. Utilization of maternal care in rural HeBei Province, the People’s Republic of China: individual and structural characteristics. Health Policy. 2004;70(2):197–206. 10.1016/j.healthpol.2004.03.001 15364149

[13] Noor A, Zurovac D, Hay S, Ochola S, Snow R. Defining equity in physical access to clinical services using geographical information systems as part of malaria planning and monitoring in Kenya. Tropical Medicine & International Health. 2003;8(10):917–26. 14516303 10.1046/j.1365-3156.2003.01112.x PMC2912492

[14] Zegeye K, Gebeyehu A, Melese T. The role of geographical access in the Utilization of institutional delivery service in rural Jimma Horro District, Southwest Ethiopia. Prim Health Care. 2014;4(1):2167–1079.1000150.

[15] Ketende C, Gupta N, Bessinger R. Facility-level reproductive health interventions and contraceptive use in Uganda. International family planning perspectives. 2003:130–7. 10.1363/ifpp.29.130.03 14519590

[16] Chamla DD, Olu O, Wanyana J, Natseri N, Mukooyo E, Okware S, et al Geographical information system and access to HIV testing, treatment and prevention of mother-to-child transmission in conflict affected Northern Uganda. Conflict and Health. 2007;1(1):12. 18053189 10.1186/1752-1505-1-12 PMC2228274

[17] Entwisle B, Rindfuss RR, Walsh SJ, Evans TP, Curran SR. Geographic information systems, spatial network analysis, and contraceptive choice. Demography. 1997;34(2):171–87. 9169275

[18] Chen S, Guilkey DK. The effect of facility characteristics on choice of family planning facility in rural Tanzania: MEASURE Evaluation, Carolina Population Center, University of North Carolina at Chapel Hill Chapel Hill, NC; 2002.

[19] Gabrysch S, Campbell OM. Still too far to walk: literature review of the determinants of delivery service use. BMC pregnancy and childbirth. 2009;9(1):34. 19671156 10.1186/1471-2393-9-34 PMC2744662

[20] THE DHS Program. MEASURE DHS SPA Methodology [cited 01/06/2019. Available from: https://dhsprogram.com/What-We-Do/Survey-Types/SPA-Methodology.cfm.

[21] The DHS Program. MEASURE DHS Methodology—Collecting Geographic Data [cited 01/06/2019. Available from: https://dhsprogram.com/what-we-do/gps-data-collection.cfm.

[22] Worku AG, Yalew AW, Afework MF. Factors affecting utilization of skilled maternal care in Northwest Ethiopia: a multilevel analysis. BMC international health and human rights. 2013;13(1):20. 23587369 10.1186/1472-698X-13-20 PMC3639034

[23] Babalola S, Fatusi A. Determinants of use of maternal health services in Nigeria-looking beyond individual and household factors. BMC pregnancy and childbirth. 2009;9(1):43. 19754941 10.1186/1471-2393-9-43 PMC2754433

[24] Kruk ME, Galea S, Prescott M, Freedman LP. Health care financing and utilization of maternal health services in developing countries. Health Policy and Planning. 2007;22(5):303–10. 10.1093/heapol/czm027 17681975

[25] Parkhurst JO, Penn-Kekana L, Blaauw D, Balabanova D, Danishevski K, Rahman SA, et al Health systems factors influencing maternal health services: a four-country comparison. Health Policy. 2005;73(2):127–38. 10.1016/j.healthpol.2004.11.001 15978956

[26] Tegegne TK, Chojenta C, Loxton D, Smith R, Kibret KT. The impact of geographic access on institutional delivery care use in low and middle-income countries: Systematic review and meta-analysis. PLoS ONE. 2018;13(8):e0203130 10.1371/journal.pone.0203130 30161201 PMC6117044

[27] Paxton A, Bailey P, Lobis S, Fry D. Global patterns in availability of emergency obstetric care. International Journal of Gynecology & Obstetrics. 2006;93(3):300–7. 16682039 10.1016/j.ijgo.2006.01.030

[28] Hong R, Montana L, Mishra V. Family planning services quality as a determinant of use of IUD in Egypt. BMC Health Serv Res. 2006;6(1):79. 16792810 10.1186/1472-6963-6-79 PMC1553443

[29] Sepehri A, Sarma S, Simpson W, Moshiri S. How important are individual, household and commune characteristics in explaining utilization of maternal health services in Vietnam? Soc Sci Med. 2008;67(6):1009–17. 10.1016/j.socscimed.2008.06.005 18635302

[30] Gabrysch S, Cousens S, Cox J, Campbell OM. The influence of distance and level of care on delivery place in rural Zambia: a study of linked national data in a geographic information system. PLoS Med. 2011;8(1):e1000394 10.1371/journal.pmed.1000394 21283606 PMC3026699

[31] Central Statistical Agency (CSA) [Ethiopia], ICF. Ethiopia Demographic and Health Survey 2016. Addis Ababa, Ethiopia, and Rockville, Maryland, USA: CSA and ICF; 2016.

[32] Ethiopian Public Health Institute, ICF International. Ethiopia Service Provision Assessment Plus (ESPA+) Survey 2014. Addis Ababa, Ethiopia and Rockville, Maryland USA: Ethiopian Public Health Institute and ICF International; 2014.

[33] Natural Earth. Free vector and raster map data [cited 31/05/2019. Available from: https://www.naturalearthdata.com/downloads/10m-cultural-vectors/.

[34] World Health Organization. Service availability and readiness assessment (SARA): an annual monitoring system for service delivery: reference manual. 2015.

[35] World Health Organization. Service Availability and Readiness Assessment (SARA): an annual monitoring system for service delivery.: Health Statistics and Information Systems, WHO; 2013.

[36] Anselin L. Local indicators of spatial association—LISA. Geographical analysis. 1995;27(2):93–115.

[37] O'Brien L. Point Pattern Analysis.(Scientific Geography Series, vol. 8). JSTOR; 1989.

[38] ESRI. How Spatial Autocorrelation (Global Moran's I) works [cited 01/06/2019. Available from: http://pro.arcgis.com/en/pro-app/tool-reference/spatial-statistics/h-how-spatial-autocorrelation-moran-s-i-spatial-st.htm.

[39] Anselin L, Sridharan S, Gholston S. Using exploratory spatial data analysis to leverage social indicator databases: the discovery of interesting patterns. Social Indicators Research. 2007;82(2):287–309.

[40] ESRI. Incremental Spatial Autocorrelation [cited 01/06/2019. Available from: http://desktop.arcgis.com/en/arcmap/10.3/tools/spatial-statistics-toolbox/incremental-spatial-autocorrelation.htm.

[41] Getis A, Ord JK. The analysis of spatial association by use of distance statistics. Geographical analysis. 1992;24(3):189–206.

[42] ESRI. How Hot Spot Analysis (Getis-Ord Gi*) works [cited 01/06/2019. Available from: http://pro.arcgis.com/en/pro-app/tool-reference/spatial-statistics/h-how-hot-spot-analysis-getis-ord-gi-spatial-stati.htm.

[43] MacKellar F. Early mortality data: sources and difficulties of interpretation. The Cambridge World History of Human Disease. 1993:209–13.

[44] Caldas de Castro M, Singer BH. Controlling the false discovery rate: a new application to account for multiple and dependent tests in local statistics of spatial association. Geographical Analysis. 2006;38(2):180–208.

[45] Ene M, Leighton EA, Blue GL, Bell BA, editors. Multilevel models for categorical data using SAS PROC GLIMMIX: the basics. SAS Global Forum 2015 Proceedings; 2015.

[46] Tom A, Bosker TASRJ, Bosker RJ. Multilevel analysis: an introduction to basic and advanced multilevel modeling: Sage; 1999.

[47] Bailey PE, Keyes EB, Parker C, Abdullah M, Kebede H, Freedman L. Using a GIS to model interventions to strengthen the emergency referral system for maternal and newborn health in Ethiopia. International Journal of Gynecology & Obstetrics. 2011;115(3):300–9. 21982854 10.1016/j.ijgo.2011.09.004

[48] Mwaikambo L, Wilkes B, Jayarajan N. Mapping: A Skill for the Future: Measure Evaluation; 2014 [cited 01/06/2019. Available from: https://measureevaluation.wordpress.com/2014/07/30/mapping-a-skill-for-the-future/.

[49] Chirwa TF, Mantempa JN, Kinziunga FL, Kandala JD, Kandala N-B. An exploratory spatial analysis of geographical inequalities of birth intervals among young women in the Democratic Republic of Congo (DRC): a cross-sectional study. BMC pregnancy and childbirth. 2014;14(1):271. 25117879 10.1186/1471-2393-14-271 PMC4139614

[50] Kruk ME, Mbaruku G, McCord CW, Moran M, Rockers PC, Galea S. Bypassing primary care facilities for childbirth: a population-based study in rural Tanzania. Health policy and planning. 2009;24(4):279–88. 10.1093/heapol/czp011 19304785

[51] Tobler WR. Cellular geography Philosophy in geography: Springer; 1979 p. 379–86.

[52] Ebener S, Guerra-Arias M, Campbell J, Tatem AJ, Moran AC, Johnson FA, et al The geography of maternal and newborn health: the state of the art. International journal of health geographics. 2015;14(1):19. 26014352 10.1186/s12942-015-0012-x PMC4453214

[53] Bailey TC, Gatrell AC. Interactive spatial data analysis: Longman Scientific & Technical Essex; 1995.

[54] Fischer M, Scholten H, Unwin D. The Moran scatterplot as an ESDA tool to assess local instability in spatial associaton. Spatial analytical perspectives on GIS. 1996.

[55] Burgert C. Spatial interpolation with demographic and health survey data: key considerations. DHS Spatial Analysis Reports. 2014;9.

[56] Rosero-Bixby L. Spatial access to health care in Costa Rica and its equity: a GIS-based study. Soc Sci Med. 2004;58(7):1271–84. 10.1016/S0277-9536(03)00322-8 14759675

[57] Getis A, Ord JK. Local spatial statistics: an overview. Spatial analysis: modelling in a GIS environment. 1996;374:261–77.

[58] Kurtz T, Link R, Tukey JW, Wallace D. Short-cut multiple comparisons for balanced single and double classifications: Part 1, Results. Technometrics. 1965;7(2):95–161. 5858969

[59] Tukey JW. The philosophy of multiple comparisons. Statistical science. 1991:100–16.

[60] Getis A, Ord J, editors. Seemingly independent tests: addressing the problem of multiple simultaneous and dependent tests. 39th Annual Meeting of the Western Regional Science Association Kauai, Hawaii; 2000.

[61] Rogerson PA. A statistical method for the detection of geographic clustering. Geographical Analysis. 2001;33(3):215–27.

[62] Julian MW. The consequences of ignoring multilevel data structures in nonhierarchical covariance modeling. Structural Equation Modeling. 2001;8(3):325–52.

[63] Moerbeek M. The consequence of ignoring a level of nesting in multilevel analysis. Multivariate Behavioral Research. 2004;39(1):129–49. 10.1207/s15327906mbr3901_5 26759936

[64] Wampold BE, Serlin RC. The consequence of ignoring a nested factor on measures of effect size in analysis of variance. Psychological Methods. 2000;5(4):425 11194206 10.1037/1082-989x.5.4.425

[65] Goldstein H. Multilevel Statistical Models 3rd edition (Arnold, London). 2003.

[66] Nich C, Carroll K. Now you see it, now you don't: A comparison of traditional versus random-effects regression models in the analysis of longitudinal follow-up data from a clinical trial. Journal of Consulting and Clinical Psychology. 1997;65(2):252 9086688 10.1037//0022-006x.65.2.252

[67] Bliese PD. An introduction to multilevel modeling techniques. Personnel Psychology. 2000;53(4):1062.

[68] Raudenbush SW, Bryk AS. Hierarchical linear models: Applications and data analysis methods: Sage; 2002.

[69] Hox JJ, Moerbeek M, Van de Schoot R. Multilevel analysis: Techniques and applications: Routledge; 2017.

